# Multicolor emission from intermediate band semiconductor ZnO_1−x_Se_x_

**DOI:** 10.1038/srep44214

**Published:** 2017-03-13

**Authors:** M. Welna, M. Baranowski, W. M. Linhart, R. Kudrawiec, K. M. Yu, M. Mayer, W. Walukiewicz

**Affiliations:** 1 Department of Experimental Physics, Faculty of Fundamental Problems of Technology, Wrocław University of Science and Technology, Wybrzeze Wyspianskiego 27, 50-370, Wroclaw, Poland; 2 Laboratoire National des Champs Magnétiques Intenses, UPR 3228, CNRS-UGA-UPS-INSA, Grenoble and Toulouse, France; 3 Materials Sciences Division, Lawrence Berkeley National Laboratory, Berkeley, California, 94720, USA; 4 Department of Physics and Materials Science, City University of Hong Kong, Kowloon, Hong Kong

## Abstract

Photoluminescence and photomodulated reflectivity measurements of ZnOSe alloys are used to demonstrate a splitting of the valence band due to the band anticrossing interaction between localized Se states and the extended valence band states of the host ZnO matrix. A strong multiband emission associated with optical transitions from the conduction band to lower E_−_ and upper E_+_ valence subbands has been observed at room temperature. The composition dependence of the optical transition energies is well explained by the electronic band structure calculated using the ***kp*** method combined with the band anticrossing model. The observation of the multiband emission is possible because of relatively long recombination lifetimes. Longer than 1 ns lifetimes for holes photoexcited to the lower valence subband offer a potential of using the alloy as an intermediate band semiconductor for solar power conversion applications.

Alloying is a well established and widely used method to engineer properties of semiconductor materials for specific applications. The properties of the alloys composed of materials that do not differ significantly from each other in terms of atom size and/or electronegativity can be predicted by simple interpolations between properties of the end-point materials. In most cases, such “well matched” alloys are easy to synthesize in the whole composition range. In contrast, alloys of materials with considerably different atom size and/or electronegativity have limited solubility and are difficult to synthesize. It has been shown previously that the electronic structure of such highly mismatched alloys (HMAs) is determined by an anticrossing interaction between localized states of the minority and extended band states of the majority component of the alloy^1^,^2^,^3^. Thus, substitution of highly electronegative minority atoms on majority atom sites, e.g. N substituting As in GaAs, leads to an anticrossing interaction between localized level of N and the conduction band of the GaAs host matrix. On the other hand, minority atoms substituting more electronegative majority atoms, e. g. As substituting N in GaN, results in an anticrossing interaction between localized level of As and the valence band of GaN. Cases of both types of interactions were demonstrated in group III–V and II–VI compound semiconductors^1^,^2^,^4^,^5^,^6^,^7^,^8^,^9^,^10^,^11^,^12^. As applied to a variety of HMAs, the electronic band structure of such materials is well described by the band anticrossing (BAC) model. The conduction band BAC model is simple as it involves only two sets of double degenerate s-symmetry states whereas the valence band BAC involves two sets of six-fold degenerate p-symmetry states. The simplicity of the conduction band BAC allowed accurate prediction of the electronic band structure and practical realization of optoelectronic devices. Thus, the BAC-induced splitting of the conduction band has been observed experimentally and was used to demonstrate intermediate band solar cells in dilute group III–V nitrides and dilute group II–VI oxides^13^,^14^,^15^,^16^.

Despite numerous studies of the HMAs with valence band anticrossing the experimental evidence of the interaction has been limited to the observed rapid band gap reduction associated with a upward shift of the upper valence band and large abrupt change in the spin orbit splitting^7^,^8^,^9^,^10^. So far, there has been no experimental observation of the predicted splitting of the valence band of HMAs. In this letter we report direct observation of simultaneous multicolor emission originating from transitions between conduction band and the subbands of ZnOSe valence band split by the BAC interaction. The observation is possible because of unexpectedly long lifetimes of holes excited to the lower subband. The origin of observed photoluminescence peaks is further confirmed by photoreflectance spectroscopy measurements. The experimental results are in excellent agreement with ***kp*** calculations (combined with the BAC interaction) of ZnOSe valence band structure. The results have important consequences for potential applications of highly mismatched alloys for intermediate band solar cells that can better utilize full solar spectrum and overcome the Schockley-Queisser limit for single junction solar cells^17^.

Phase separation has been previously observed in III–V and II–VI HMAs. While the solubility limits of N in GaAs or O in ZnSe (or ZnTe) are known to be in the impurity limit of 10^18^–10^19^/cm^3^, dilute alloys with up to 10% can be grown using highly non-equilibrium growth methods such as low temperature molecular beam epitaxy (LTMBE)^18^,^19^ or ion implantation with pulsed laser melting^1^,^20^. Moreover, GaNAs over the entire composition has been grown using LTMBE method^21^. In the present study growth of ZnOSe HMAs was accomplished using pulsed laser deposition (PLD) method at a substrate temperature of ~200 °C which is much lower than typical growth of high quality ZnO films (~>800 °C). It has been shown before that the amount of Se incorporated in ZnO gradually decreases with increasing substrate temperature^22^. To avoid phase separation the study has been limited to samples with low composition of <10% of Se. Our XRD measurements showed that films with Se content >10% exhibited “amorphous like” structure^22^.

## Results and Discussion

**Figure 1:**
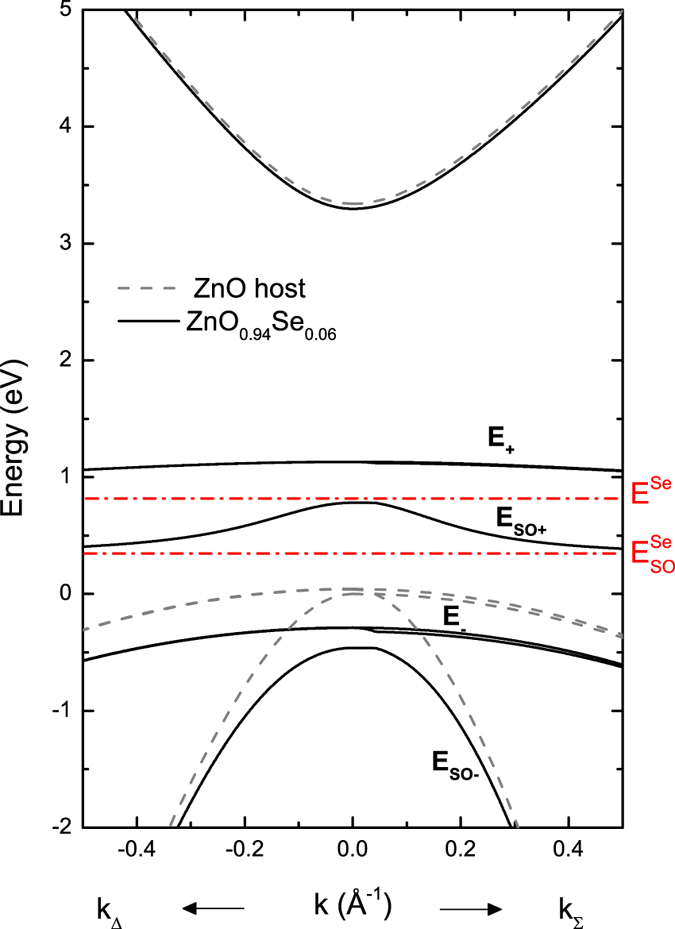
Band structure close to the Γ point of the Brillouin zone for ZnO_1−x_Se_x_ with x = 0.06, calculated using the valence band anticrossing model (solid line). The dashed line illustrates the band dispersion of ZnO host material. The Se impurity level and its spin orbit are also shown in this figure by the red dash-dot lines.

The effects of the BAC interaction on the electronic band structure of cubic HMAs with the p-symmetry valence have been previously considered to describe optical properties of GaAsN^23^ as well as GaAsSb^8^ and GaSbBi^8^ alloys. The interaction between the six-fold degenerate valence band and the six localized p-states of the minority element leads to 12 × 12 ***kp*** Hamiltonian. However, because ZnO crystallizes in a wurtzite crystal structure, we use a Hamiltonian developed by Suzuki and Uenoyama^24^ to describes the three highest-lying valence bands and interaction with the conduction band in ZnOSe. The BAC interaction is included between localized p-like states of Se and the host valence bands. The Se localized energy level used in the calculations is taken as ~0.9 eV above the ZnO valence band maximum (VBM) at room temperature^11^. Additionally, it is important to note that the Se p-like states have a large spin orbit splitting of 0.37 eV^25^ that has to be included in the calculations. Figure 1 shows an example of the calculated band structure close to the center of the Brillouin zone for ZnOSe with 6% of Se. According to these calculations the anticrossing interaction between localized Se states and extended states of ZnO host splits the valence band and leads to the formation of several subbands: E_+_ and E_SO+_ above the host matrix valence band edge and two subbands E_−_ and E_SO−_ below the ZnO valence band. Note that the spin orbit splitting for Se-derived E_+_ subbands is much larger than for ZnO-derived E_−_ subbands. The dispersion relations in Fig. 1 indicate that the large energy separation between E_+_ and E_−_ subbands offers a potential to observe multiple optical transitions in this alloy system.

**Figure 2:**
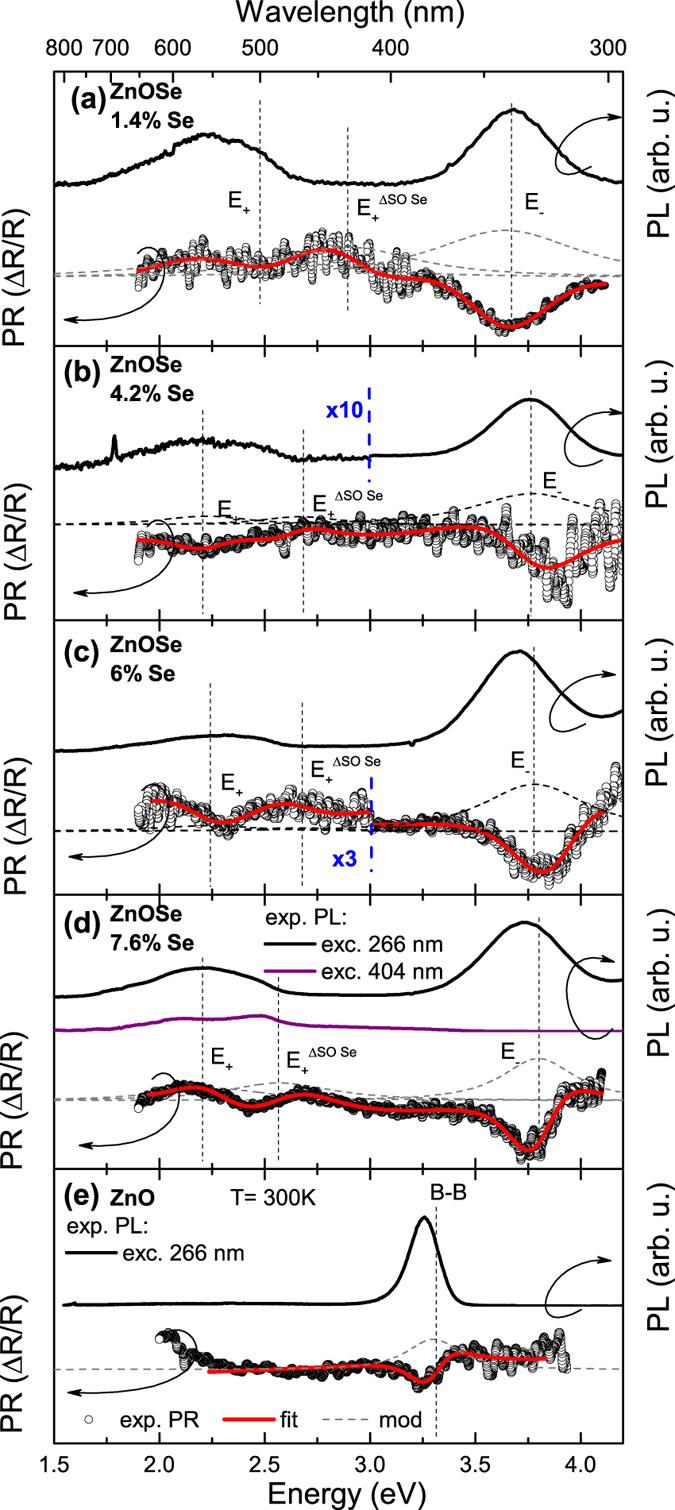
Representative PR and PL spectra of ZnOSe alloys. (**a**) PL and PR spectra of the ZnO_0.986_Se_0.014_ layer. PL was measured under 266 nm (black line) excitation wavelength. The PR spectrum is marked by open circles. The red line is a fitting curve according to Aspnes formula^27^ with three resonances. The dashed gray lines are moduli of PR resonances with the maxima corresponding to the energies of optical transitions. (**b**) PL and PR spectra of the ZnO_0.958_Se_0.042_ layer. (**c**) PL and PR spectra of the ZnO_0.94_Se_0.06_ layer. (**d**) PL and PR spectra of the ZnO_0.924_Se_0.076_ layer. Additional PL spectrum under 404 nm excitation is also shown (violet line). (**e**) PL and PR spectra of the ZnO reference film.

In order to confirm the predicted BAC induced splitting of the valence band we have synthesized a series of ZnO_1−x_Se_x_ layers with x < 0.08. The composition and thickness of the ZnOSe samples were measured by Rutherford backscattering spectrometry (RBS) using a 3.04 MeV alpha beam^11^. The accuracy of the composition obtained by RBS is estimated to be ~±5%. Figure 2a–d shows the results of PL and PR measurements performed on a ZnO_0.986_Se_0.014_, ZnO_0.958_Se_0.042_, ZnO_0.94_Se_0.06_, and ZnO_0.924_Se_0.076_ samples, respectively (similar spectra were obtained for the rest of the samples). Two distinct PL bands are observed under the 266 nm laser excitation. For the ZnO_0.924_Se_0.076_ sample the high energy peak is located at ~3.6 eV and the low energy asymmetric peak at about 2.3 eV. The energies of these peaks coincide quite well with transitions from the conduction band edge (CBE) to E_−_ (3.6 eV) and the two transitions from CBE to E_+_ and CBE to E_SO+_ (~2.5 eV) shown in Fig. 1. As is seen in Fig. 2(d) the excitation with lower photon energy CW 404 nm (3.07 eV) laser does not (as expected) produce the high energy PL peak but results in a clearly visible splitting of the lower energy PL into two bands. The energy separation of ~0.3 eV between those two low energy peaks is near the expected value (0.37 eV) of the energy difference between transitions from CBE to E_+_ and from CBE to E_SO+_ marked in Fig. 1. This indicates that we were able not only to observe the splitting between E_−_ and E_+_ subbands but also to resolve the transitions between the spin orbit split bands.

To further ascertain the origin of the observed emissions we performed PR measurements represented by open circles in Fig. 2a–d. Photoreflectance features corresponding to the PL bands are clearly observed. This confirms that strong absorption between extended band-like states is responsible for the transitions and excludes the possibility that deep defect states could be responsible for the low energy PL^26^. A detailed analysis of the PR spectra using the critical point model^27^ reveals that the low energy feature is composed of two resonances that are denoted as E_+_ and E_SO+_ bands. The fitting curve is presented as a red line and the moduli of each transition as grey dashed lines. Maxima of the moduli indicate the transition energies. Dashed vertical lines are drawn to represent the energy of each transition. The observed redshift of the PL peaks relative to the PR resonances can be attributed to the local potential fluctuation which are a characteristic feature of most HMAs^28^,^29^,^30^,^31^,^32^,^33^,^34^.

As is seen in Fig. 2(e) a Se-free ZnO reference film grown under the same conditions has only one high energy PL peak and corresponding to it one PR resonance, both related to the direct band gap transition^35^. These results clearly show that Se is responsible for the low energy emission observed in ZnOSe alloys.

**Figure 3:**
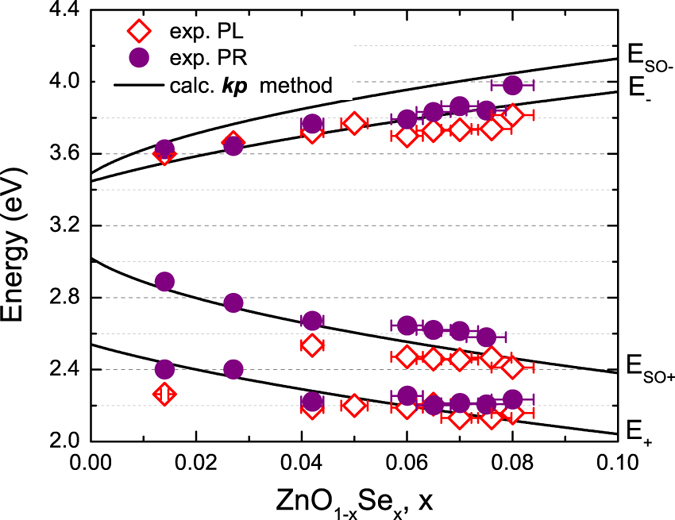
The PR (closed circles) and PL (opened circles) transitions as a function of Se content for ZnOSe samples grown on GaAs (closed circles). The solid lines depict the evolution of the transitions between the conduction and E_−_, E_SO+_, E_+_ valence subbands as a function of Se content, as modeled using the valence BAC model.

Figure 3 shows composition dependencies of the optical transition energies determined from PL and PR measurements along with the calculations based on ***kp*** BAC model. The uncertainty in the determination of the transitions energies is mostly associated with the spectra fitting procedure and is about 10 meV, while the alloy composition is established with about 5% uncertainty. The calculated transition energies are in a very good agreement with experiment assuming that the localized Se level is located at 0.85 eV above ZnO VBE and the BAC model coupling parameter equals to 2.4 eV. The Se level energy is in a good agreement with previously used value of 0.9 eV^11^. However, the presently determined coupling constant is two times larger than 1.2 eV of ref. 11. The difference originates from the fact that the previous work used a simplified VBAC model which ignored spin degeneracy effects in ZnOSe alloys. An interesting feature of the results shown in Fig. 3 is the much larger spin orbit splitting of the E_+_ subband compared to that of the E_−_ subband. This reflects the fact that the E_SO+_ subband is mostly derived from Se states which have a spin orbit splitting of 0.37 eV, whereas the E_SO-_ subband originates from ZnO valence band that has spin orbit splitting of only 0.016 eV^36^.

**Figure 4:**
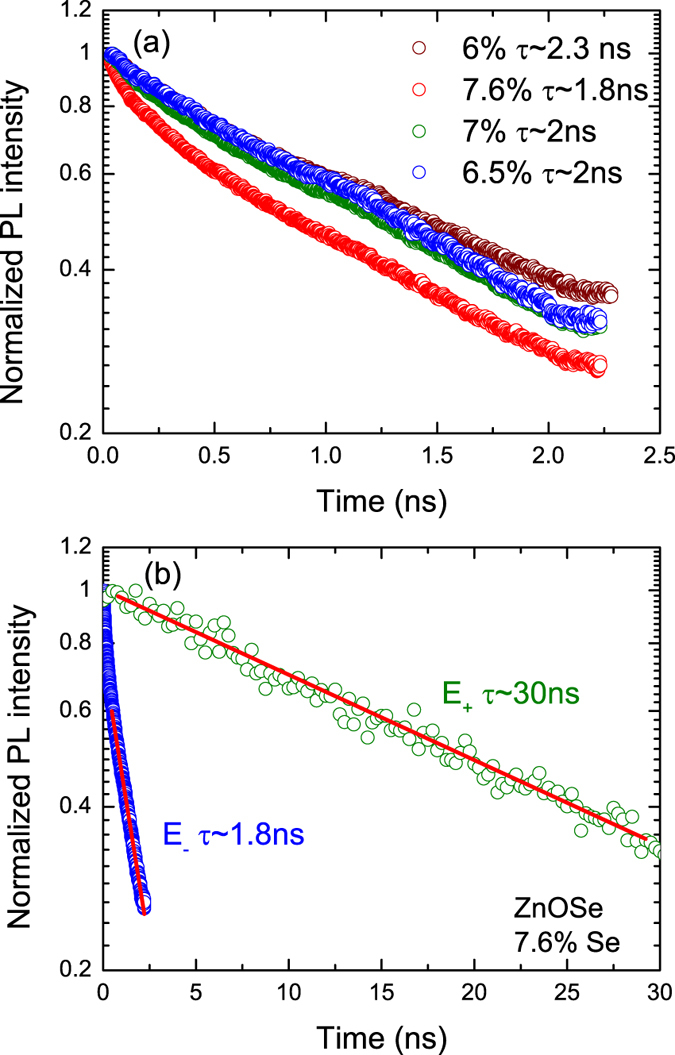
(**a**) PL decay curves at peak maximum obtained from TRPL measurements corresponding to the E_−_ transition for different Se compositions. (**b**) PL decay curves at peak maximum obtained from TRPL measurements for sample with 7.6% of Se corresponding to the E_−_ (blue open circles) and E_+_ (green open circles) transitions. The fitting is marked as red lines with the assumption of exponential decay.

The successful observation of the strong and well resolved high energy PL associated with transitions between CBE and E_−_ subbands is surprising as the holes excited to this low lying subband have large variety of nonradiative recombination paths to higher energy subbands. To determine the hole lifetimes we have performed TRPL measurements. Time decays of the PL at maximum intensity are shown in Fig. 4. The lifetimes were determined from exponential fitting of the experimental data. The unexpectedly long lifetime of almost 2 ns found for the E_−_ transitions indicates low nonradiative recombination rates for holes excited to the E_−_ subband. Similar lifetime values for E_−_ transitions were obtained for different Se compositions. Representative spectra for different Se compositions are shown in Fig 4(a). As expected, a significantly longer lifetime of 30 ns is observed for the transitions to the top valence subband Fig 4(b). One of strongest and often used argument against the viability of HMAs as materials for intermediate band solar cell was that electrons excited to the upper subband in the conduction band or holes excited to the lower subband in the valence band will have exceedingly short lifetimes and could not be efficiently collected. Our present results show that it does not always have to be the case and that lifetimes in excess of ns are possible.

## Conclusions

In conclusion, a simultaneous multicolor emission involving valence band split by BAC interaction has been observed in ZnOSe HMA. The three separate PL emission peaks are attributed to transitions from the CBE to E_+_, E_SO+_ and E_−_ subbands resulting from the splitting of the valence band of ZnO by the anticrossing interaction with localized Se states. The results are well explained by combining ***kp*** and BAC models of the electronic band structure of ZnOSe. TRPL measurements show exceptionally long lifetimes of almost 2 ns for the E_−_ and 30 ns for E_+_ transitions. Such long lifetimes of photoexcited holes offer a potential of using highly mismatched alloys as absorbers in solar power conversion devices.

## Methods

The layers were grown on sapphire substrates by PLD technique. The details of samples growth and results of structural characterization can be found in refs 11,37. Photoluminescence (PL) measurements were performed using two different laser excitation sources: (i) 266 nm 1 kHz pulse laser (pulse duration ~1 ns, average power 1 mW) and (ii) 404 nm CW laser. The PL spectrum was detected by a Si CCD detector. Time resolved photoluminescence (TRPL) measurements were performed using the third harmonic of a Ti:Sapphire laser at 266 nm. The PL signal was dispersed by a 0.3 m focal length monochromator and detected by Hamamatsu streak camera with S-20 photocathode. A tungsten halogen lamp was used in photoreflectance (PR) measurements. Photomodulation was provided by a 266 nm laser beam. Reflected light was analyzed by a single grating 0.55 m monochromator and detected by a photomultiplier tube. The phase-sensitive detection of the PR signal was made using a lock-in amplifier. All of the measurements were performed at room temperature.

## Additional Information

**How to cite this article**: Welna, M. *et al*. Multicolor emission from intermediate band semiconductor ZnO_1−x_Se_x_. *Sci. Rep.* **7**, 44214; doi: 10.1038/srep44214 (2017).

**Publisher's note:** Springer Nature remains neutral with regard to jurisdictional claims in published maps and institutional affiliations.
